# Pharmacokinetics of cucurbitacin B from *Trichosanthes cucumerina* L. in rats

**DOI:** 10.1186/s12906-019-2568-7

**Published:** 2019-07-04

**Authors:** Natthaphon Hunsakunachai, Nitra Nuengchamnong, Weena Jiratchariyakul, Tanawan Kummalue, Phisit Khemawoot

**Affiliations:** 1 Department of Pharmacology and Physiology, Faculty of Pharmaceutical Sciences, Chulalongkorn University, Pathumwan, Bangkok, Thailand; 2 Science Laboratory Centre, Faculty of Science, Naresuan University, Tha Pho, Phitsanulok, Thailand; 3 Department of Pharmacognosy, Faculty of Pharmacy, Mahidol University, Thung Phayathai, Ratchathewi, Bangkok, Thailand; 4 Department of Clinical Pathology, Faculty of Medicine Siriraj Hospital, Bangkok Noi, Bangkok, Thailand; 5 Preclinical Pharmacokinetics and Interspecies Scaling for Drug Development Research Unit, Chulalongkorn University, Pathumwan, Bangkok, Thailand

**Keywords:** Cucurbitacin B, *Trichosanthes cucumerina*, Cucurbitaceae, Pharmacokinetics

## Abstract

**Background:**

Cucurbitacin B is the major bioactive constituent in *Trichosanthes cucumerina* L. fruits, which the pharmacological properties have been studied for decades particularly an anti-tumor activity. The pharmacokinetic profile of this compound is still limited and investigation is needed for further phytopharmaceutical product development. This study aimed to investigate the pharmacokinetic profile of cucurbitacin B after administering the compound at different doses and routes to rats.

**Methods:**

Male Wistar rats (*n* = 6) were treated by cucurbitacin B extracted from *Trichosanthes cucumerina* L. The cucurbitacin B was administered at 0.1 mg/kg intravenously or by oral gavage at 2–4 mg/kg. Blood samples and internal organs were collected serially within 24 h after administration. Urine and feces were collected from time 0 to 48 h. The level of cucurbitacin B in biological samples was determined by liquid chromatography-tandem mass spectrometry.

**Results:**

The absolute oral bioavailability of cucurbitacin B was approximately 10%. The maximum concentration in plasma after normalization by dose ranged from 4.85–7.81 μg/L and the time to reach maximum value was approximately within 30 min after oral dosing. The level of cucurbitacin B in plasma increased proportionally to the given dose. After intravenous administration, cucurbitacin B had a large volume of distribution of about 51.65 L/kg and exhibited a high tissue to plasma concentration ratio, approximately 60 to 280-fold in several organs. Negligible amount of unchanged cucurbitacin B could be detected in urine and feces and accounted less than 1% of administered dose.

**Conclusion:**

Cucurbitacin B had low oral bioavailability, but could be distributed extensively into internal organs with a high volume of distribution and tissue to plasma ratio. Only negligible amounts of unchanged cucurbitacin B were excreted via urine and feces suggesting that the compound might be biotransformed before undergoing an excretion. Further studies of the metabolic pathway and tissue uptake mechanism are required to strategize the future development of cucurbitacin B into clinical studies.

## Background

**Fig. 1:**
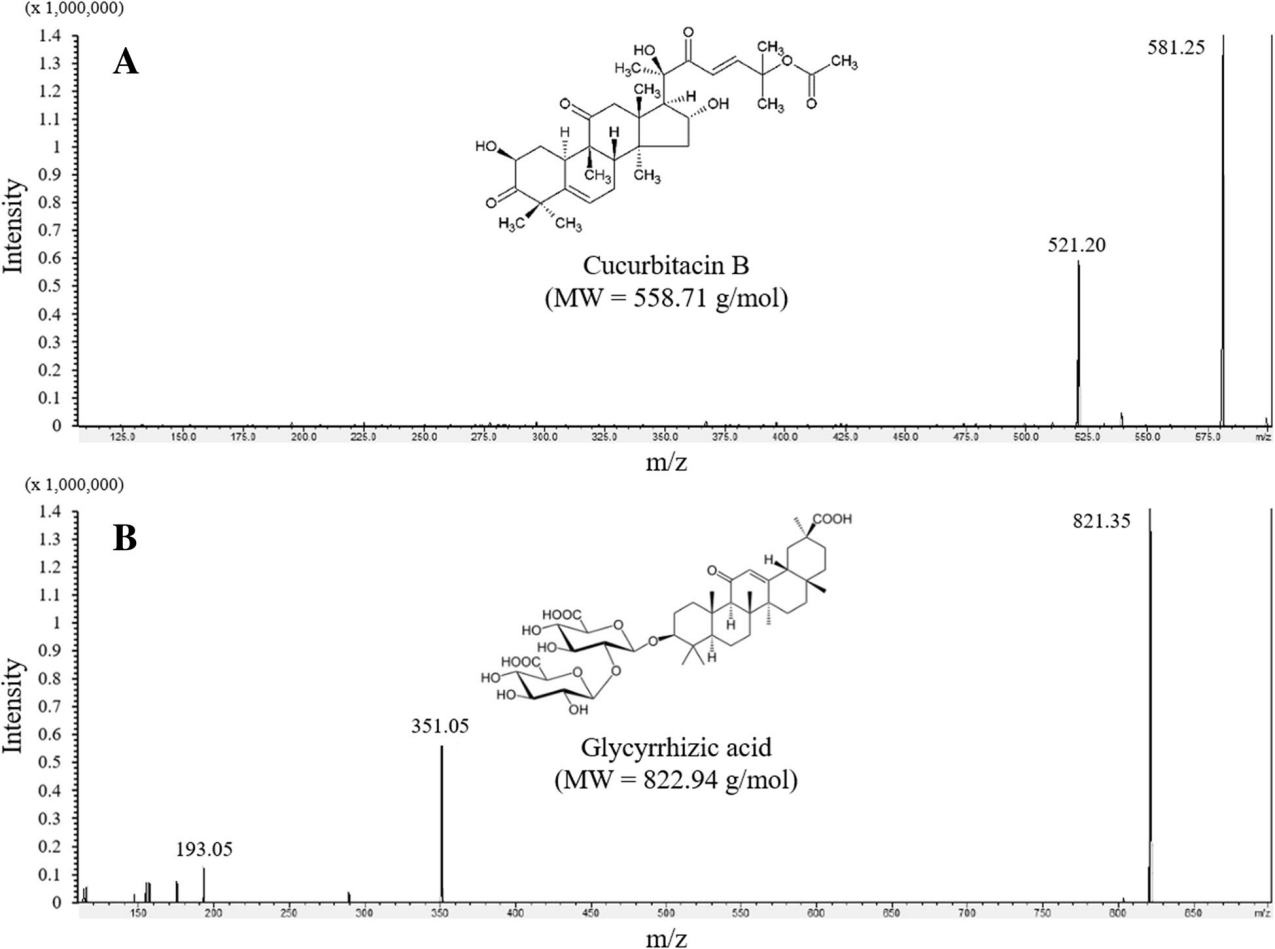
Product ion scanning of parent ions and fragmentation patterns for cucurbitacin B (**a**) and glycyrrhizic acid, internal standard (**b**)

*Trichosanthes cucumerina* L. is a monoecious annual herb belonging to the family of Cucurbitaceae [1]. *T. cucumerina* has been used in traditional medicine, especially in India. It was reported that *T. cucumerina* could be used for different ailments in a part-dependent manner e.g., using their fruits for skin swelling or purgative activity, their leaves for antipyretic or antispasmodic effect [1, 2]. The major bioactive components in *T. cucumerina* that possess these pharmacological properties have been investigated. At least 12 compounds were found in the fruit juice, especially cucurbitacin B, which was the most abundant in various parts of *T. cucumerina*. Cucurbitacin is an oxygen-rich compound that can be found mostly in cucurbitaceous plants [3]. Currently, there are at least 19 members that belong to the cucurbitacin group. Each member is slightly different in chemical structure, by which it can be identified as cucurbitacin A to cucurbitacin T [4]. Cucurbitacin B is one of the most studied bioactive components from *T. cucumerina* [5]. The chemical structure of cucurbitacin B is demonstrated in Fig. 1.

Cucurbitacin B is a tetracyclic triterpenic molecule that possesses high lipophilicity. The molecular mass of this compound is 558.71 g/mol and it is slightly soluble in water with XlogP 2.60. Various reports confirmed the pharmacological activity of cucurbitacin B. It has been extensively studied in anti-inflammatory [6–8], anti-atherosclerotic [9], hepatoprotective [10], and particularly antineoplastic activity [4]. The mechanisms of action of cucurbitacin B for anti-cancer properties have also been studied for decades; the main mechanisms involved proapoptosis by STAT3 pathway inhibition [11, 12]. Moreover, cucurbitacin B could promote cell cycle arrest by mediating multiple signaling pathways, for example, it could induce G2/M cell cycle arrest in breast cancer cells [13, 14] and lung cancer cell line [15].

Pharmacokinetic investigations of cucurbitacin B have been conducted in animal models. Zhao and colleagues investigated rats administered cucurbitacin B at the dose of 20 mg/kg in 0.5% CMC. They found that cucurbitacin B reached the highest concentration within 1.75 h with an elimination half-life of approximately 2.5 h. Cucurbitacin B was able to be distributed well into various organs, as a result of large volume of distribution, but the evidence supporting concentration in tissue was inadequate [16]. Furthermore, there was no proposed metabolic pathway of cucurbitacin B in vitro or in vivo. Therefore, the objective of this study was to investigate the pharmacokinetic profile of cucurbitacin B after oral and intravenous administration in which the tested doses were varied to examine the pharmacokinetic profile. The intravenous and oral doses were selected as 0.1 mg/kg and 2–4 mg/kg, respectively based on antineoplastic activity [17, 18]. Additionally, tissue kinetics and route of excretion were also investigated. The results obtained from this study will provide useful information in understanding the in vivo pharmacokinetics of cucurbitacin B and can used as a basis to optimize an appropriate dosage regimen for further study.

## Methods

### Chemicals

Cucurbitacin B for the pharmacokinetic experiment was supplied by Dr. Weena Jiratchariyakul, Faculty of Pharmacy, Mahidol University (Bangkok, Thailand), which was extracted from *T. cucumerina* fruit juice to obtain the purity of 90.7%. Analytical standard of cucurbitacin B (≥ 97%) and glycyrrhizic acid, an internal standard, were purchased from Sigma-Aldrich Corporation (St. Louis, United States). Isoflurane, an anesthetic agent was acquired from Indochina Healthcare Limited (Bangkok, Thailand).

### Animals

Eight weeks old male Wistar rats were purchased and delivered from the National Laboratory Animal Centre, Mahidol University (Bangkok Thailand). They were housed under controlled conditions until their weight was approximately 400–500 g. The controlled temperature in the animal housing room was maintained at 24 ± 2 °C, and humidity ranged from 40 to 60%. Lighting in the housing room was also regulated with a 12-h light/dark cycle, and rats were allowed to access food and water ad libitum. Rats were divided randomly into designated groups and food was restricted for 12 h before cucurbitacin B administration. The number of animals per each experimental group was six, which was calculated by G*power version 3.1.9.2. (Kiel, Germany). All animal related experiments were conducted under the protocol approved by the ethical committee of the Faculty of Pharmaceutical Sciences, Chulalongkorn University (Bangkok, Thailand). The approved protocol review number is 16–33-002, which was accepted on 9th June 2017.

### Blood chemistry

Liver and kidney function were assessed prior to the pharmacokinetic experiment. Biochemical markers for aspartate aminotransferase (AST), alanine aminotransferase (ALT), and creatinine were used to determine these functions. Plasma was collected before and 24 h after cucurbitacin B administration. Measurement of these markers was conducted by Professional Laboratory Management Corporation (Bangkok, Thailand). AST and ALT were quantified by kinetic method according to IFCC recommendations, whereas creatinine level in plasma was measured by using Cobas 6000 automated analyzer in enzymatic method (chemiluminescence).

### Pharmacokinetic experiment

Cucurbitacin B solution in DMSO/water for injection was administered to the rats at different doses and routes (intravenous cucurbitacin B at 0.1 mg/kg, and oral cucurbitacin B at 2–4 mg/kg). All cucurbitacin B solution given to the animals was prepared freshly prior to the experiment. Each rat was anesthetized with 10% isoflurane by chamber induction method before intravenous administration or blood collection. Both approaches were conducted via lateral tail vein. The dose of cucurbitacin B for injection was calculated based on amount of cucurbitacin B in the solution to obtain an equivalent dose of 0.1 mg/kg. In the case of oral administration, a gavage needle was used to supply the pre-specified dose of cucurbitacin B at 2, and 4 mg/kg. For blood collection, pre-heparinized needles and syringes were used during the blood sampling process. Approximately 300 μL of blood sample was collected serially after cucurbitacin B administration at different time points as follows: 0.083 (5 min), 0.25 (15 min), 0.5 (30 min), 1, 2, 4, 8, 16, and 24 h. Collected blood samples were then centrifuged at 3000×*g* for 10 min to obtain plasma, and stored at − 20 °C until further process. After each blood sampling session, the rat was placed in the metabolic cage, then urine and feces were segregated and collected automatically by the functionality of cages at distinct periods of time comprehensive of 0–24, and 24–48 h. Measurement of urine volume and feces weight were completed right after sample collection, then they were stored at − 20 °C until analysis.

### Internal organ collection

Heart, lungs, liver, stomach, spleen, small intestine, kidneys, and brain were collected after intravenous administration of cucurbitacin B 0.1 mg/kg injection. Euthanization was performed by 10% isoflurane with prolonged exposure at 1, 2, and 4 h after administration, and death was confirmed by exsanguination. Before organ removal, 1 mL of blood was withdrawn by cardiac puncture procedure, centrifuged at 3000×*g* for 10 min to obtain plasma, and stored at − 20 °C until further analysis. Unnecessary connective tissue was removed from the isolated organs by surgical blade and scissor. Excessive blood within the removed organs was washed twice by 0.9% saline solution at the temperature below 4 °C.

### Sample pretreatment

Protein precipitation was the extraction procedure used in this study. In brief, plasma 50 μL was treated with 200 μL of methanol containing 25 ng of glycyrrhizic acid as the internal standard. The samples were mixed for 10 min and then centrifuged at 10,000×*g* for 10 min. One hundred fifty microliter of supernatant from each sample was collected for further LC-MS/MS analysis.

For urine samples, the volume of each sample was measured, mixed for 10 min, centrifuged at 3000×*g* for 10 min and each supernatant was collected. Fifty microliter of supernatant was mixed with 200 μL of methanol containing 25 ng of glycyrrhizic acid. The final mixture was centrifuged at 10,000×*g* for 10 min and 150 μL of supernatant from each sample was collected for further LC-MS/MS analysis.

The weight of each feces sample was measured, homogenized, and adjusted to the total volume of 10 mL by methanol. Centrifugation at 3000×*g* for 10 min was performed and then supernatant was collected from each sample. Fifty microliter of supernatant was mixed with 200 μL of methanol containing 25 ng of glycyrrhizic acid. The final mixture was centrifuged at 10,000×*g* for 10 min and 150 μL of supernatant from each sample was collected for further LC-MS/MS analysis.

Internal organs including heart, lungs, liver, stomach, spleen, small intestine, kidneys, mammary glands, and brain were weighed out in 50 mg aliquots and then mixed with 200 μL of methanol containing 25 ng of glycyrrhizic acid. The mixture was later homogenized in an ice bath for 10 min and then centrifuged at 10,000×*g* for 10 min. One hundred fifty microliter of supernatant from each sample was collected for further LC-MS/MS analysis.

### Instrumentation

LC-MS/MS was used to determine cucurbitacin B level in biological samples. Nexera UHPLC (Kyoto, Japan) consisting of a vacuum degasser, a binary pump, an autosampler, and a column thermostat equipped with Shimadzu 8060 LCMS (Kyoto, Japan) was applied for this experiment. Control of both liquid chromatography and mass spectrometer was operated by LabSolution version 5.86 software (Kyoto, Japan). The mobile phase used in the system comprised of 0.2% v/v formic acid in water (pH 2.5), and methanol. Gradient phase elution was performed in every sample analysis. The ratio of mobile phase was changed as follows: starting with 45% methanol then gradually increasing to 90% methanol at 2.5 min, this ratio of methanol was maintained until 3 min, then constantly decreased to 45% methanol at 3.5 min, and this proportion was continued until 5 min. The summary of gradient eluting pattern is demonstrated in Table 1. The flow rate used in the system was 0.3 mL/min with an injection volume of 15 μL. C18 reversed phase column, model Phenomenex Synergi Fusion-RP (Torrance, United States) equipped with Guard C18 column, model Phenomenex SecurityGuard Fusion-RP (Torrance, United States) was used for compound separation process. Column oven was maintained at 40 °C throughout the analytical procedure.

**Table 1:** The gradient eluting pattern for cucurbitacin B determination by using LC-MS/MS.

Time (min)	Methanol (%)	0.2% v/v Formic acid (%)
0.00	45	55
2.50	90	10
3.00	90	10
3.50	45	55
5.00	45	55

**Fig. 2:**
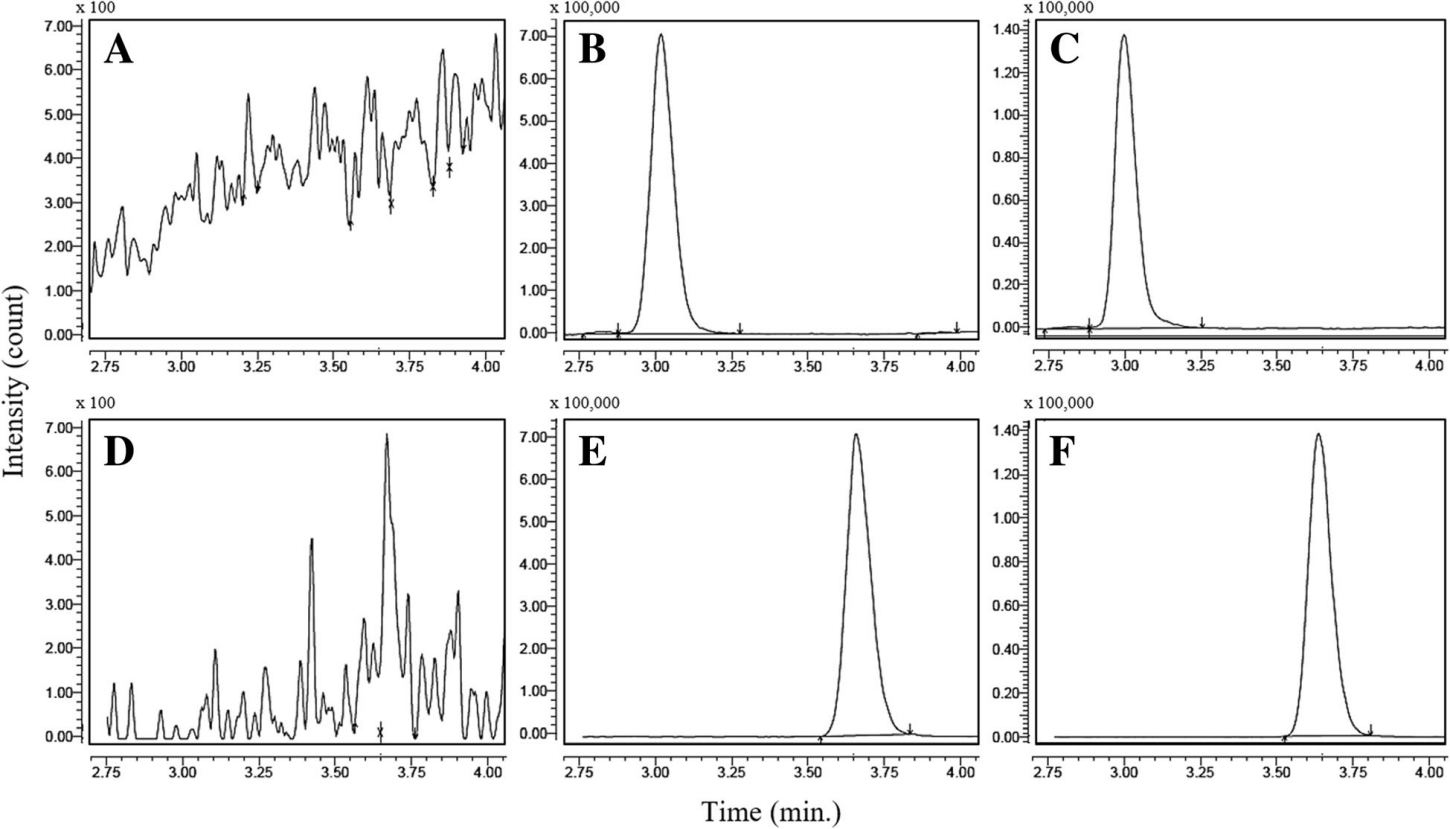
Representative chromatograms of blank rat plasma after monitoring under cucurbitacin B condition (**a**); blank plasma with cucurbitacin B at 5 μg/L (**b**); cucurbitacin B in an unknown plasma sample (**c**); blank rat plasma after monitoring under glycyrrhizic acid condition (**d**); pre-spiked blank plasma with glycyrrhizic acid at 25 ng (**e**); glycyrrhizic acid added in an unknown plasma sample (**f**)

The mass spectrometry parameters were optimized to obtain an appropriate condition for cucurbitacin B quantification. In brief, positive and negative ionization mode was applied for cucurbitacin B and glycyrrhizic acid measurements, respectively. Multiple reaction monitoring (MRM) was used to perform mass spectrometric quantification of analyte and internal standard. MS/MS spectra of cucurbitacin B at m/z of 559.32 [M + H]^+^ was not able to be detected and it displayed relatively low intensity compared to m/z of 581.25 [M + Na]^+^. Accordingly, the [M + Na]^+^ ion was chosen for the cucurbitacin B as the precursor ion. The ion transition of cucurbitacin B and glycyrrhizic acid was defined as m/z 581.25/521.20 and m/z 821.25/350.90, respectively. The MS/MS spectra of cucurbitacin B and glycyrrhizic acid were shown in Fig. 1. Collision energy used in the system was − 26.9 and 40.5 V applied for cucurbitacin B and glycyrrhizic acid, respectively. Interface condition was defined as follows: nebulizing gas flow: 3 L/min; heating gas flow: 10 L/min; interface temperature: 300 °C; heating block temperature: 400 °C; and drying gas flow: 10 L/min. Retention time of cucurbitacin B and glycyrrhizic acid was 3.01 and 3.65 min, respectively. Representative chromatograms of cucurbitacin B and glycyrrhizic acid are shown in Fig. 2.

### Method validation

The analytical method was performed according to US FDA bioanalytical method validation - guidance for industry [19]. The following parameters were evaluated during the validation process including: selectivity, matrix effect and recovery, linearity, lower limit of quantification (LLOQ), accuracy, precision, and stability. Quality control (QC) samples were prepared prior to the validation process and three concentrations of analytical cucurbitacin B standard solution including 0.5, 10, and 15 μg/L were selected as representatives of low, medium, and high concentration, respectively.

Selectivity was assessed by comparing the chromatograms of six different batches of blank rat plasma with the cucurbitacin B spiked plasma samples (un-extract). Recovery and matrix effects were evaluated by comparing the peak area of cucurbitacin B spiked in post-extraction plasma (extract) at concentrations of QC with peak area obtained from standard solutions.

Linearity was evaluated by constructing the calibration curve. Range of concentration in each analyte was determined by the results obtained from the preliminary study. The number of calibration points was designed to be around 7 to 8 points. Each sample was quantified by calculating the peak area ratio (y) and known concentration of each analyte (x). Calibration equation and correlation coefficient (R^2^) were also calculated with least squares linear regression analysis. The lowest concentration of the calibration curve was defined as the lower limit of quantitation or LLOQ. At this point, the chromatographic peak area was required to be at least 5 times higher than baseline and both calculated precision and accuracy needed to be no more than ±20%.

Accuracy was calculated in term of relative error (% RE), which compared the value of the concentration obtained from the calibration equation with each known analyte concentration. Intra-assay, and inter-assay precision were evaluated by comparing peak areas of six replicates of extracted QC samples on three different days.

The stability test consisted of short term stability at room temperature, long term stability at storage conditions, freeze-thaw stability, and autosampler stability. Short term stability was evaluated at room temperature for 24 h. Long term stability was assessed at − 20 °C for 3 months. The freeze–thaw stability of cucurbitacin B was tested by analyzing QC at three different concentrations subjected to three freeze-thaw cycles (− 20 and 25 °C). Lastly, autosampler stability was determined by measuring the peak area obtained from freshly prepared QC samples compared with samples that were stored in the autosampler compartment for 24 h.

### Data analysis

Biochemical markers including AST, ALT, and creatinine were presented as mean ± standard deviation (SD). Comparison of these markers between pre- and post-treatment groups was conducted by paired sample t-test in which statistical difference was decided at *p*-value < 0.05. Cucurbitacin B plasma levels obtained from LC-MS/MS analysis were further analyzed by constructing the curve between the calculated concentrations (μg/L) and blood sampling time points (h). Cucurbitacin B plasma concentrations in the plasma concentration-time curve were reported as mean ± SD. Pharmacokinetic parameters were computed by PK solutions 2.0 software (Montrose, United States) with non-compartmental analysis. The pharmacokinetic parameters were listed as follows: maximum plasma concentration (C_max_), time to maximum plasma concentration (T_max_), area under the curve from time zero to last sampling time point (AUC_0-t_), area under the curve from time zero to infinity (AUC_0-inf_), absolute oral bioavailability (F), elimination half-life (t_1/2_), volume of distribution (V_d_), clearance (CL), and mean residence time (MRT). Absolute oral bioavailability was calculated according to the following equation:

$$ \%\mathrm{F}=\frac{{\mathrm{AUC}}_{0-\operatorname{inf}}\mathrm{after}\ \mathrm{oral}\ \mathrm{administration}}{{\mathrm{AUC}}_{0-\operatorname{inf}}\ \mathrm{after}\ \mathrm{IV}\ \mathrm{administration}}\times \frac{\mathrm{IV}\ \mathrm{dose}}{\mathrm{oral}\ \mathrm{dose}}\times 100 $$

All of these pharmacokinetic parameters were presented as mean ± SD. For the tissue distribution study, the extent of cucurbitacin B that resided in each organ was depicted as tissue to plasma concentration ratio (K_app_). This parameter was computed by comparing concentration of cucurbitacin B in each internal organ to that obtained from plasma with the identical time point. Comparison of each parameter from different doses and routes of cucurbitacin B was determined by Mann-Whitney U test, because the data possessed non-normal distribution. All statistical analyses were performed by using IBM SPSS Statistics version 22 (Armonk, United States).

## Results

### Method validation

Selectivity was determined by analyzing the potential interference that eluted simultaneously with cucurbitacin B and glycyrrhizic acid, the internal standard. As illustrated in Fig. 2, there was no significant chromatographic peak of interference, which eluted at the same retention times as cucurbitacin B (3.01 min) and glycyrrhizic acid (3.65 min). We concluded that the method provided a good selectivity to determine the cucurbitacin B level in biological samples.

The calibration curves were constructed in the range of 0.1–20 μg/L. Linearity of analytical method was determined by least square linear regression by using 1/x^2^ as a weighting factor. The typical linear equation was y = 1.0055x – 0.0408 with R^2^ = 0.999 for cucurbitacin B. Accuracy of each concentration used in the regression line was limited within the range of ±15%, except for the LLOQ, which was acceptable within ±20%. The LLOQ was 0.1 μg/L for cucurbitacin B.

Accuracy was evaluated at three different concentration of quality control sample including 0.5, 10, and 15 μg/L. Relative errors (%RE) were calculated by comparing the value obtained from the measurement with the known concentration. The results were summarized in Table 2, and showed the relative errors (RE) of three quality control concentrations were within the acceptable range of ±15%. Intra-assay and inter-assay precision was assessed for three consecutive days. The results are demonstrated in Table 2 and show that the coefficient of variation (CV) of the intra- and inter-assay in three concentrations was acceptable within ±15%. These results allowed us to conclude that both accuracy and precision of this analytical method were suitable for cucurbitacin B determination.

**Table 2:** The precision and accuracy for cucurbitacin B determined by LC-MS/MS.

QC concentration (μg/L)	Precision (%CV)		Accuracy (%RE)	
	Within-batch	Between-batch	Within-batch	Between-batch
0.5	6.31	5.77	5.40	1.64
10	2.19	3.75	−1.60	−4.14
15	2.50	2.28	−8.38	−7.84

*CV* Coefficient of variation, *RE* Relative error

Recoveries of cucurbitacin B are demonstrated in Table 3, and revealed that the recoveries of cucurbitacin B after protein precipitation varied from 73.14–79.73%. This suggested that there were some matrix effects affecting the cucurbitacin B measurement. This range of recoveries was still acceptable because they provided similar recovery patterns at every concentration of quality control.

**Table 3:** The recovery of cucurbitacin B after extraction from rat plasma

Sample Number	Concentration of Cucurbitacin B (μg/L)					
	LQC 0.5 μg/L		MQC 10 μg/L		HQC 15 μg/L	
	Un-extract	Extract	Un-extract	Extract	Un-extract	Extract
1	0.79	0.59	11.72	9.57	18.77	14.15
2	0.63	0.51	12.69	9.83	18.71	13.93
3	0.67	0.50	12.48	9.51	18.77	13.88
4	0.61	0.52	12.52	10.10	18.69	13.86
5	0.64	0.52	12.39	9.70	18.92	13.39
6	0.62	0.52	11.96	10.02	18.89	13.26
Mean	0.66	0.53	12.29	9.79	18.79	13.74
SD	0.07	0.03	0.37	0.24	0.09	0.35
%CV	10.25	6.31	3.01	2.45	0.50	2.51
Absolute Recovery	79.73		79.61		73.14	

*QC* Quality control, CV, coefficient of variation; RE, relative error.

Stability of cucurbitacin B was tested in various conditions and the results are shown in Table 4. The stability of this compound ranged from 81.30–109.50% depending on the test condition. The room temperature stability, when the quality control samples were left at 25 °C for 24 h, seemed to have the lowest recovery compared to the other test conditions (ranged from 81.30–86.10%). This suggested that the biological samples should be prepared freshly within 24 h. In other test conditions, they provided satisfactory recoveries, which ranged from 90.50–109.50%.

**Table 4:** The stability of cucurbitacin B at different storage conditions

Conditions	QC concentration (μg/L)	Measured values (μg/L)	SD	%CV	%RE	%Recovery
Room temperature (12 h)	0.5	0.43	0.02	4.90	14.20	81.34
	10	8.20	0.19	2.34	18.01	83.32
	15	11.80	0.15	1.25	21.35	86.16
Storage at −20 °C (3 months)	0.5	0.48	0.03	5.66	4.60	90.54
	10	9.81	0.65	6.58	1.86	99.72
	15	13.46	0.42	3.10	10.29	97.93
Three freeze-thaw cycles	0.5	0.49	0.03	6.39	3.00	92.01
	10	9.79	0.22	2.21	2.13	99.50
	15	13.68	0.46	3.34	8.81	99.54
Autosampler (10 °C)	0.5	0.47	0.03	7.25	6.20	89.05
	10	10.78	0.76	7.01	−7.78	109.57
	15	13.84	0.39	2.82	7.71	100.75

*QC* Quality control, *CV* Coefficient of variation, *RE* Relative error

### Animal tolerability

All animals tested with cucurbitacin B showed a good tolerability after administration for 24 h. The general appearance of animals was observed throughout the experiment without significant changes in behavior or physical abnormality. Only soft stool in some animals could be detected in high dose administered group. Liver and kidney function of animals were evaluated with plasma biochemical markers as illustrated in Table 5. For liver function, AST and ALT were measured and compared between pre- and post-dosing for each intervention. The results demonstrated that after treatment, neither AST nor ALT changed significantly compared to prior treatment. AST level seemed to increase slightly in the animals given 0.1 mg/kg intravenous dose and 4 mg/kg oral dose. The minimal change in this enzyme appeared to be not significant for the clinical status of the animals, as these values were still within the normal range. Likewise, creatinine level, used to determine kidney function was not altered after cucurbitacin B administration for 24 h.

**Table 5:** General appearance and biochemical markers indicating liver and kidney functions before and after cucurbitacin B treatment

Parameters	Cucurbitacin B 0.1 mg/kg IV		Cucurbitacin B 2 mg/kg PO		Cucurbitacin B 4 mg/kg PO	
	Pre-dosing (0 h)	Post-dosing (24 h)	Pre-dosing (0 h)	Post-dosing (24 h)	Pre-dosing (0 h)	Post-dosing (24 h)
General appearance	Normal	Normal	Normal	Normal	Normal	Normal
AST (U/L)	35.40 ± 4.15	49.20 ± 3.27	36.67 ± 1.53	32.33 ± 11.68	33.50 ± 8.10	50.25 ± 0.96
ALT (U/L)	11.40 ± 6.31	11.00 ± 3.94	11.00 ± 1.00	6.33 ± 1.53	16.75 ± 6.50	12.25 ± 5.62
Creatinine (mg/dL)	0.22 ± 0.03	0.23 ± 0.02	0.20 ± 0.02	0.19 ± 0.03	0.23 ± 0.02	0.22 ± 0.03

The data represent as mean ± SD (*n* = 6). AST, aspartate aminotransferase; ALT, alanine aminotransferase

### Plasma concentration-time curve and pharmacokinetic parameters

**Fig. 3:**
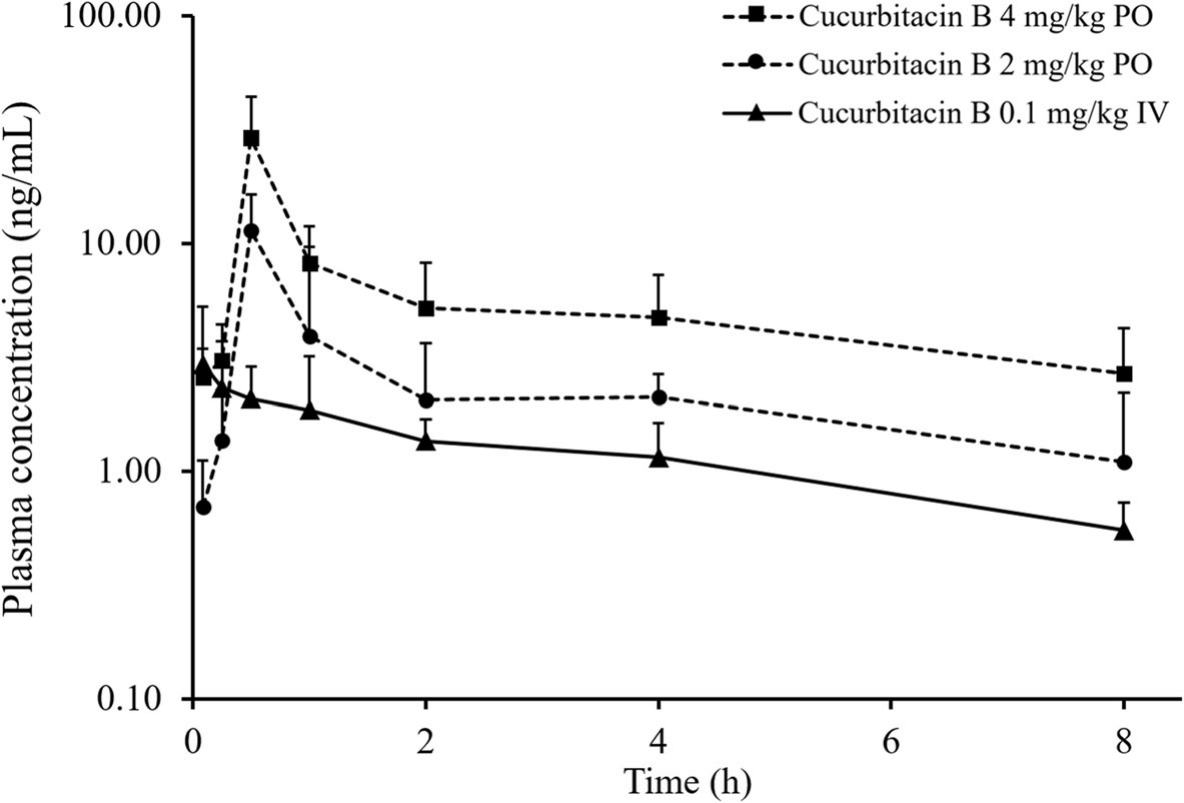
Mean plasma concentration-time curves of cucurbitacin B after intravenous administration at 0.1 mg/kg (−▲-) and oral administration at 2 mg/kg (−●-) and 4 mg/kg (−■-). Each data point represents as mean ± SD

Plasma concentration-time profile after cucurbitacin B intravenous injection is shown in Fig. 3. The pharmacokinetic parameters of cucurbitacin B after each dose and route of administration were calculated and are displayed in Table 6. After intravenous administration, cucurbitacin B showed a very high volume of distribution (V_d_ = 51.65 ± 39.16 L/kg) but possessed a short half-life (t_1/2_ = 5.08 ± 2.87 h). This indicated that cucurbitacin B was distributed thoroughly in the animal body and was cleared out of the plasma within a short period of time. It was concordant with the computed clearance (CL) in which the value was approximately 7.24 L/h/kg.

**Table 6:** Pharmacokinetic parameters of cucurbitacin B

Parameters	Cucurbitacin B 0.1 mg/kg IV	Cucurbitacin B 2 mg/kg PO	Cucurbitacin B 4 mg/kg PO
C_max_ (μg/L)	N/A	9.70 ± 3.95	31.24 ± 10.50
T_max_ (h)	N/A	0.50 ± 0.00	0.60 ± 0.22
AUC_0-t_ (μg·h/L)	13.92 ± 11.11	15.10 ± 3.57	45.22 ± 10.14
AUC_0-inf_ (μg·h/L)	17.95 ± 13.21	25.33 ± 12.13	52.42 ± 29.58
Normalized AUC_0-inf_ (μg·h/L)	N/A	12.67 ± 6.07	13.11 ± 7.40
F (%)	N/A	10.25 ± 7.29	10.25 ± 5.63
V_d_ (L/kg)	51.65 ± 39.16	N/A	N/A
CL (L/h/kg)	7.24 ± 2.92	N/A	N/A
t_1/2_ (h)	5.08 ± 2.87	N/A	N/A
MRT (h)	6.03 ± 2.93	9.95 ± 12.27	5.50 ± 2.28

The values represent as mean ± SD (n = 6). Cmax, maximum plasma concentration; T_max_, time to maximum plasma concentration; AUC_0-t_, area under the curve from time zero to last sampling time point; *AUC*_*0-inf*_ Area under the curve from time zero to infinity, *F* Absolute oral bioavailability, *V*_*d*_ Volume of distribution, *CL* Clearance, *t*_*1/2*_ Elimination half-life, *MRT* Mean residence time

As demonstrated in Fig. 3, all plasma concentration-time profiles of orally administered cucurbitacin B possessed similar patterns, although the curve of cucurbitacin B after the 4 mg/kg dose was slightly higher than that of 2 mg/kg. Cucurbitacin B concentration in plasma after oral doses reached the highest concentration within 1 h when the maximum plasma concentration (C_max_) was approximately 9.70 and 31.24 μg/L in 2 and 4 mg/kg dosing, respectively. The cucurbitacin B concentration gradually decreased in both oral doses to 1.09 and 2.66 μg/L within 8 h and could not be further detected at 16 h (below LLOQ). Calculated AUC_0-inf_ of cucurbitacin B after 4 mg/kg oral dosing was 2-fold higher than that of 2 mg/kg, thus AUC_0-inf_ needed to be adjusted by including the dose into the formula. Comparison of normalized AUC_0-inf_ was performed and the result showed that there was no statistical difference between 2 and 4 mg/kg oral dosing.

The computed absolute bioavailability was approximately 10% in both oral doses. Comparison of the absolute bioavailability from each group was performed and no statistical difference between 2 mg/kg and 4 mg/kg oral dosing was detected. This suggested that different doses did not affect the extent of absorption of cucurbitacin B.

### Tissue distribution

**Fig. 4:**
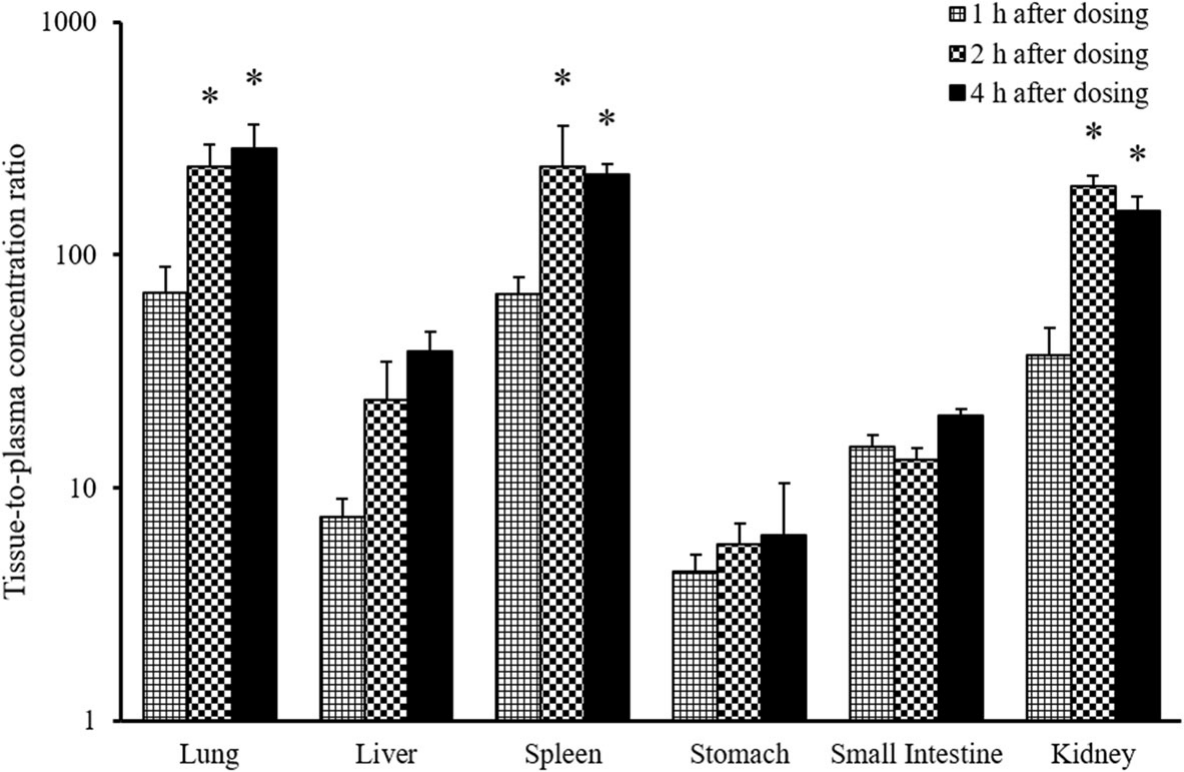
Mean tissue to plasma concentration ratio of cucurbitacin B after intravenous administration at 0.1 mg/kg. The data are represented as mean ± SD. * *p*-value < 0.05 compared to the mean tissue-to-plasma concentration ratio at 1 h after cucurbitacin B administration

Cucurbitacin B showed distribution potential in various organs as shown in Fig. 4. The compound could penetrate to the organs extensively when tissue to plasma concentration ratio (K_app_) was determined. The organs that displayed the highest K_app_ included lung, spleen, and kidney, respectively. It appeared that cucurbitacin B concentrations in both lung and spleen could increase about 60 times higher than in plasma at 1 h after intravenous injection and continually accumulate to around 240 times at 2 h. The K_app_ of cucurbitacin B in lung and spleen remained unchanged at 4 h post-injection compared to the 2 h indicating that cucurbitacin B could be deposited in these organs for a long period of time after intravenous administration. Kidney was another organ that showed a good distribution site despite a slightly lower K_app_ compared to spleen and lung. Other organs of interest, such as brain, heart, liver, stomach, and small intestine were also investigated. Only negligible amounts of cucurbitacin B could be detected in brain and heart, but could not be quantified because the concentration was lower than the LLOQ. Lesser ratios of cucurbitacin B in liver, stomach, and small intestine were investigated. Only 4–14 times K_app_ were observed at 1 h after administration. Interestingly, only the ratio found in liver could accumulate to 23.72 and 38.29 in 2 and 4 h, respectively. In contrast, the ratios in stomach and small intestine were not changed dependently with time. The K_app_ in stomach slightly increased, but unfortunately, the values between each time point were not significantly different.

### Excretion study

Urine and feces collected from the animals during the experiment were quantified for the cucurbitacin B level. Negligible amounts of unchanged cucurbitacin B were removed from the animals’ bodies as shown in the Table 7. Less than 1% of unchanged cucurbitacin B could be detected in excreta after administration for 0–24 and 24–48 h.

**Table 7:** The percent recovery of unchanged cucurbitacin B in excreta compared to administered dose

Recovery (%)	Cucurbitacin B 0.1 mg/kg IV	Cucurbitacin B 2 mg/kg PO	Cucurbitacin B 4 mg/kg PO
Urine at 0-24 h	< 1.00	< 1.00	< 1.00
Urine at 24-48 h	< 1.00	< 1.00	< 1.00
Feces at 0-24 h	< 1.00	< 1.00	< 1.00
Feces at 24-48 h	< 1.00	< 1.00	< 1.00

n = 6

## Discussion

Cucurbitacin B possessed a wide variety of pharmacological activities including anti-inflammatory, anti-atherosclerotic, hepatoprotective, and especially anti-cancer activity [10]. Additional study of pharmacokinetic profiles could provide more information in understanding the relationship between the presented amount of the compound in systemic circulation or targeted organs to their therapeutic effects. In the current study, the pharmacokinetics of cucurbitacin B were investigated. The dose of cucurbitacin B used in this study was decided, based on anti-neoplastic activity. From the tolerability test, the results showed that animals could well tolerate the given doses. There were no significant changes in behavior or physical appearance. The AST and ALT levels indicating liver function were also not affected when compared to baseline. Even though the AST level was increased in 0.1 mg/kg intravenous and 4 mg/kg oral dosing, it did not alter the clinical status of the animals since the values were still within the normal range. ALT level might be a better biochemical marker to determine liver function resulting from higher specificity [20]. Kidney function was determined by using creatinine as a biomarker and the results demonstrated that there was no difference between pre- and post-dosing. From these outcomes, we confirmed that cucurbitacin B did not alter the liver or kidney function, which are important metabolic organs for xenobiotic biotransformation and excretion.

Pharmacokinetics profiling was initiated by analyzing the plasma concentration-time curves. After intravenous administration, the curve was plotted on semi-log scale and it was found that cucurbitacin B follows a one compartment pharmacokinetic model. Least squares linear regression analysis was applied to confirm this hypothesis and the result showed a good correlation coefficient with R^2^ = 0.951. The elimination half-life was calculated from intravenous data and the value was approximately 5.08 ± 2.87 h. The previously reported half-life was about 2.50–3.19 h [16, 21], which seemed lower than the current study. This might be due to the different ages and conditions of rodents used in the study [22, 23]. The volume of distribution was also computed from the intravenous curve and was approximately 51.65 ± 39.16 L/kg. It could be predicted that cucurbitacin B had high possibility of distributing to several tissues, resulting in only a trivial amount detected in plasma. For oral administration, cucurbitacin B achieved the maximum concentration within 30 min, which means this compound could be absorbed rapidly by the gastrointestinal tract. T_max_ obtained from the current study was faster than previous reports that described the values as approximately 1.75–2.41 h [16, 21]. This could be explained by the different formulation used in the study. 40% v/v dimethylsulfoxide was selected in the current study to obtain a clear solution for both intravenous and oral preparation. Compared to suspensions prepared by carboxymethylcellulose in other studies, the clear solution was readily absorbed [24], resulting in a shorter T_max_. In spite of rapid absorption, the extent of cucurbitacin B that could reach systemic circulation was limited. This is the first study that reports the absolute oral bioavailability of cucurbitacin B. We found that only about 10% of an oral dosage could attain systemic circulation. This might be because the compound could not be completely absorbed through the intestinal wall or it was first pass metabolized by intestinal or hepatic enzymes prior moving to the systemic circulation. Interestingly, the amount of cucurbitacin B concentration found in plasma was increased proportionally to the given dose. It was characterized by the comparable normalized AUC_0-inf_ between 2 and 4 mg/kg oral dosing. This indicated that cucurbitacin B might have linear pharmacokinetics at a pharmacologically active dose range.

Tissue distribution was examined by collecting the internal organs at different time points. Tissue to plasma concentration ratio (K_app_) was calculated to determine the extent of cucurbitacin B in each organ. The results revealed that cucurbitacin B could penetrate extensively into lungs, spleen, and kidneys, respectively. This phenomenon could be described by the characteristics of these organs possessing high blood perfusion, together with the high lipophilic property of cucurbitacin B (XlogP = 2.6). This result could support the in vitro study that tested its anti-neoplastic activity in lung, and kidney cancer cell lines. Kausar and colleagues demonstrated that cucurbitacin B provided an anti-proliferative effect, which could reduce the cell viability of HCC-827 to 25% after incubation at dose of 0.25 μM for 72 h [15]. The highest concentration cucurbitacin B level found in plasma was detected at 29.21 μg/L, after 4 mg/kg oral administration, but was not enough to exert an effect. Fortunately, the concentration detected in lungs was drastically higher than that in plasma. An intravenous dose of cucurbitacin B at 0.1 mg/kg showed an appropriate level for lung cancer cells. However, the concentration presented in our study was evaluated in healthy rodents. Physiological changes and microenvironments in cancerous tissue might affect the kinetics of this compound in animal models of lung cancer. Lower K_app_ were observed in liver and small intestine, which might be explained by the biotransformation process occurring in these organs, resulting in a lower amount of cucurbitacin B. Only negligible amounts of cucurbitacin B could be detected in brains and hearts, which were lower than LLOQ. Nonetheless, cucurbitacin B could be retained in several organs at a much higher extent, and seemed to persist longer than that in plasma. Therefore, the duration of action of cucurbitacin B could not be estimated solely by elimination half-life in plasma. The concentration deposited at the active site should be taken into consideration, as it might represent better antineoplastic activity.

Excretion of unchanged cucurbitacin B was assessed by calculating the recovery compared to the given dose. We found that less than 1% of unchanged cucurbitacin B could be detected in excreta 0–48 h after dosing. This suggested that cucurbitacin B might be biotransformed prior to excretion. Currently, there was no available cucurbitacin B metabolism profile, and glucuronide conjugated cucurbitacin B was proposed as a major metabolite. Other reports mentioned that the metabolic pathway of cucurbitacin derivatives, cucurbitacin D and I, were metabolized by glucuronidation reactions [25]. Therefore, we incubated the excreta samples with β-glucuronidase from *Escherichia coli* (1000 units) for 15 min, the reaction was stopped by adding the methanol and then evaluated for unconjugated cucurbitacin B by LC-MS/MS. We found that the concentrations of cucurbitacin B obtained after β-glucuronidase reaction were not significantly different compared to without a reaction, and indicated that cucurbitacin B did not metabolized mainly via direct glucuronide conjugation. Metabolite screening was also performed by using Agilent 6540 UHD Accurate Mass TOF LC/MS (Santa Clara, United States) in positive ionization mode (data not shown). There were some interesting molecules, which were detected in urine and fecal samples including 835.2322, 319.1372, and 222.0799 Da. Precisely matching and characterizing possible metabolites will require future study. From current knowledge, we concluded that cucurbitacin B was not metabolized by direct glucuronide conjugation. The molecule might be involved in the complex processes of phase I combined with phase II enzymatic reactions.

## Conclusions

Cucurbitacin B possessed poor absolute oral bioavailability, which was approximately 10%. The compound could be distributed broadly to several organs, which could be the site of action including lungs, spleen, and kidney after intravenous administration. The major metabolic pathway of cucurbitacin B still remained unclear and the unchanged form was not found in excreta. The pharmacokinetic profile obtained from this study might be beneficial for researchers to strategize and design an appropriate dosage regimen of cucurbitacin B as an antineoplastic agent in future study.

## Data Availability

The datasets used and/or analyzed during the current study are available from the corresponding author on reasonable request.

## Abbreviations

ALT: Alanine aminotransferase
AST: Aspartate aminotransferase
AUC_0-inf_: Area under the curve from time zero to infinity
AUC_0-t_: Area under the curve from time zero to last sampling time point
CL: Clearance
C_max_: Maximum plasma concentration
CV: Coefficient of variation
DMSO: Dimethylsulfoxide
F: Absolute oral bioavailability
IFCC: The International Federation of Clinical Chemistry and Laboratory Medicine
K_app_: Plasma concentration ratio
LC-MS/MS: Liquid chromatography tandem mass spectrometry
LLOQ: Lower limit of quantitation
m/z: Mass-to-charge ratio
MRM: Multiple reaction monitoring
MRT: Mean residence time
QC: Quality control
R^2^: Correlation coefficient
RE: Relative error
SD: Standard deviation
t_1/2_: Elimination half-life
T_max_: Time to maximum plasma concentration
V_d_: Volume of distribution
XlogP: Partition coefficient
